# Disparities in Mortality Between Appalachian and non-Appalachian Regions of Kentucky

**DOI:** 10.13023/jah.0503.04

**Published:** 2023-12-01

**Authors:** Sonali S. Salunkhe, Sahal Alzahrani, Beatrice Ugiliweneza

**Affiliations:** Idaho State University; Saudi Electronic University

**Keywords:** Appalachia, drug-related mortality, health disparities, Kentucky, mortality rates, opioid-related mortality

## Abstract

**Introduction:**

In the opioid epidemic, the U.S. faces a significant public health crisis, with some areas of the country, such as rural and Appalachian regions, suffering more than others. The differential regional impact of the crisis in Kentucky—a state with both non-metropolitan/metropolitan and Appalachian/Non-Appalachian statuses—has not yet been documented despite such knowledge being essential to the success of overdose prevention efforts.

**Purpose:**

This study compares all-cause, drug- and opioid-related mortality between counties in different regions of Kentucky: Appalachian non-metropolitan, Appalachian metropolitan, non-Appalachian non-metropolitan, and non-Appalachian metropolitan.

**Methods:**

Age-adjusted mortality data from the Centers for Disease Control and Prevention’s Wide-ranging Online Data for Epidemiologic Research (CDC WONDER, 2000–2019) were used. County-level demographic and socioeconomic data were obtained from the U.S. Census Bureau, 2010 American Community Survey. Statistical analyses were performed with negative binomial regression models with a log link.

**Results:**

The Appalachian non-metropolitan region of Kentucky had a significantly higher (p < .05) all-cause mortality (1,076/100,000) compared to the state’s non-Appalachian metropolitan (904/100,000), non-Appalachian non-metropolitan (959/100,000), and Appalachian metropolitan (938/100,000) regions. Within non-Appalachian regions, non-metropolitan rates were higher than metropolitan (p = .0006). For drug- and opioid-related mortality, non-metropolitan and metropolitan regions had comparable rates within non-Appalachia, as well as within Appalachia. Appalachian regions had twice the mortality rates of non-Appalachian regions of the state (p < .05). Among the Appalachian counties, non-metropolitan counties had higher all-cause mortality than metropolitan counties.

**Implications:**

The findings from this study can help healthcare practitioners and public health officials develop interventions addressing drug-related and opioid-related mortality in Kentucky targeted to the regions where rates are significantly higher. Also, the information on geographic, demographic, and socioeconomic factors related to these types of mortality can be used to design interventions specific to the target population’s socio-demographics.

## INTRODUCTION

Increase in drug-related mortality is a pressing public health issue in the U.S.^1^ According to the Centers for Disease Control and Prevention’s (CDC) National Center for Health Statistics, a total of 91,799 drug-overdose fatalities occurred in 2020 across the U.S.^2^ An increase in drug-overdose mortality has been cited as a critical driver in decreasing life expectancy in the U.S. compared to other countries.^3^ The estimated number of opioid-involved drug poisoning deaths was 10,232 in 1999 and 39,999 in 2015—a 290.1% increase.^4^

**Figure 1:**
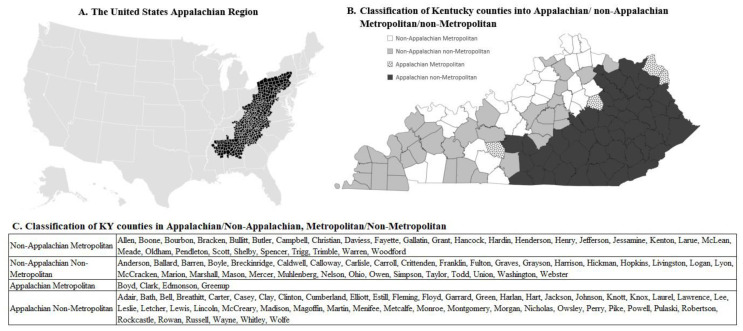
Map of the U.S. Appalachian Region (A) and classification of KY counties into Appalachian/non-Appalachian and Metropolitan/non-Metropolitan (B, C)

Although drug-overdose fatalities have risen nationally, some geographical regions have been disproportionately affected.^1^ Research suggests that rural regions have registered higher increases in narcotic drugs than urban regions,^5^ and it has been reported that Appalachian regions have higher opioid-overdose deaths than in the rest of the U.S.^6^ Specifically, the Appalachian non-metropolitan areas of the United States have been more adversely affected by the drug epidemic.^1^,^7^ In four of these Appalachian states (Fig. 1A)^1^,^8^—Kentucky, North Carolina, Virginia, and West Virginia—drug-related mortality, especially opioid-related mortality, increased dramatically from the late 1990s to the early 2000s.^1^ In Kentucky, substantial increase in drug-related mortality was noted in 2020,^9^ prompting a Health Alert Network (HAN) Advisory to expand prevention action.^10^

The differential regional impact in Kentucky, a state with both non-metropolitan/metropolitan and Appalachian/Non-Appalachian statuses, has not yet been documented despite this knowledge being essential to the success of prevention efforts. To fill this gap, the present study compares all-cause, drug-related, and opioid-related mortality rates among the Appalachian non-metropolitan, Appalachian metropolitan, non-Appalachian non-metropolitan, and non-Appalachian metropolitan regions of Kentucky.

## METHODS

### Study Outcomes

The outcomes were age-adjusted all-cause, drug-, and opioid-related mortality from mortality population estimates obtained from CDC’s WONDER platform (data spanning 2000–2019). The age-adjusted mortality values are provided as a rate per 100,000. All-cause and drug-related categories are pre-defined in CDC WONDER. Opioid-related mortality was extracted using the following ICD-10 codes: T40.0 (Opium); T40.1 (Heroin); T40.2 (Other opioids); T40.3 (Methadone); T40.4 (Other synthetic narcotics); and T40.6 (other and unspecified narcotics). The data were obtained on a county level.

### Study Groups

The outcomes between Appalachian non-metropolitan, Appalachian metropolitan, non-Appalachian non-metropolitan, and non-Appalachian metropolitan regions of Kentucky (Fig. 1B, next page) were compared. This classification was obtained using the rural–urban continuum codes from the Economic Research Service of the U.S. Department of Agriculture (USDA).^11^ Counties were classified as metropolitan and non-metropolitan and are listed in Fig. 1C (next page).

### Study Period

Mortality was evaluated between 2000 and 2019, representing 20 years of the latest CDC WONDER data. The year 2020 was not included, as there was an exceptionally high mortality rate due to the COVID-19 epidemic. The aggregated data were considered because CDC WONDER does not provide rate data on counties with fewer than 20 deaths. The goal was to maximize counties.

### County-level Covariates

Covariates were demographics and socioeconomics. Information about sex, age, race/ethnicity, and poverty composition of counties was obtained from the U.S. Census Bureau 2010 American Community Survey. For each demographic subcategory, count numbers with standard errors (SE) were provided. The year 2010 was selected, as it is the mid-study period. “Age” was grouped into five-year categories with additional estimates for 18 years and older, 21 years and older, 62 years and older, and 65 years and older. From these data, categories were calculated for 0–17 years, 18–64 years, and 65 years and older, along with their SE. The variable “Race” was categorized into white, black, American Indian and Alaskan Native (AIAN), Asian, and Native Hawaiian and Other Pacific Islander (NHOPI). Poverty was defined as the percentage of the adult population living below the federal poverty level in 2010.

### Statistical Analysis

Summary statistics were presented for each study group using a percent estimate (for covariates: sex, age, race, ethnicity, poverty) or rate per 100,000 (for outcomes: overall, drug-related, and opioid-related mortality) with associated standard deviation (SD). For covariates, the frequency count SD was calculated as ${SD}_{i}={SE}_{i}\times \sqrt{n_{i}}$ (*n_i_* being the population estimate of the county; *i* representing the county) for each county and as $SD=\sqrt{\frac{\Sigma (n-1)*{SE}_{i}^{2}}{\Sigma n_{i}-n}}$ assuming counties to be independent. SDs for the percent and rate per 100,000 figures were calculated by dividing the count SDs by 100 and 100,000, respectively. Comparisons of covariates and outcomes were obtained from a fit negative binomial regression with a log link, including the study group as the only fixed factor in the models.

**Figure 2:**
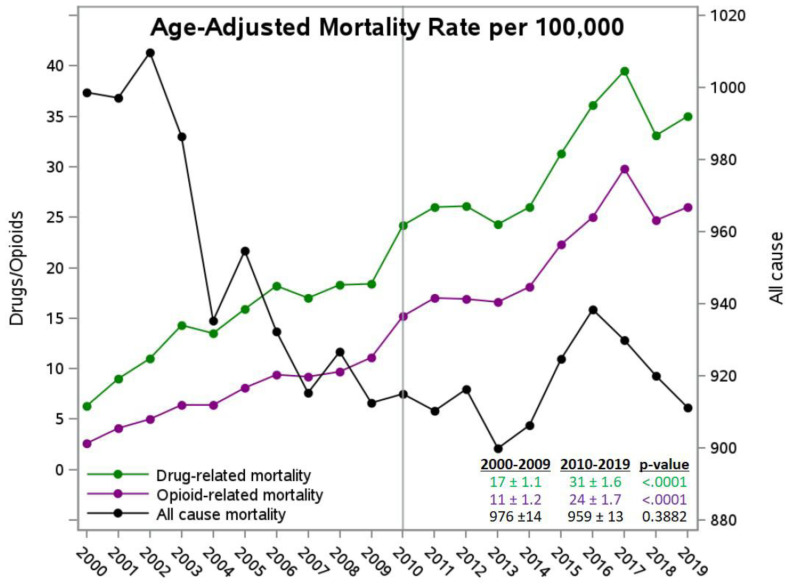
Age-adjusted mortality in KY, 2000–2019 NOTE: Circle-filled points represent age-adjusted mortality estimates over the years from CDC Wonder data. Values in the lower-right corner show age-adjusted estimates over 10-year periods and their standard errors compared with negative binomial models.

Comparisons of age-adjusted mortality between study groups were obtained by adding the region (non-Appalachian metropolitan, non-Appalachian non-metropolitan, Appalachian metropolitan, and Appalachian non-metropolitan), year period (2000–2009 and 2010–2019), and their interaction as covariates in the models. Comparisons across year periods were quantified in rate ratios (RR) f SE. All tests were two sided with a significance level set to 0.05. Statistical analyses were performed in SAS 9.4 (SAS Inc., Cary NC). Visualization of county location (Fig. 2) was obtained via Microsoft Excel 365 using the classification obtained from the USDA.^11^

### Ethical Considerations

Because this analysis used publicly available data, IRB review was not required.

## RESULTS

### Description of the Population

Of the 120 total Kentucky counties, 31 are non-Appalachian metropolitan counties (population n = 2317,627; 2010 estimates of the Commonwealth of Kentucky); 35 are non-Appalachian non-metropolitan counties (n = 785,562); 4 are Appalachian metropolitan counties (n = 133,683); and 50 are Appalachian non-metropolitan counties (n = 1,048,956). Demographic and socioeconomic information is presented in Table 1 (next page).

Across all four regions, there were comparable distributions of sex and age. The predominant race in all four regions was white, with Appalachian non-metropolitan counties having a significantly higher rate of white residents than non-Appalachian metropolitan ones (97% v. 85%, *p* = .0210). Conversely, the black population was significantly lower in Appalachian non-metropolitan (2%) compared to non-Appalachian metropolitan (12%, *p* < .0001) and non-Appalachian non-metropolitan (6%, *p* < .0001) counties. The Asian population was significantly higher in non-Appalachian metropolitan counties than across Appalachian non-metropolitan ones (2% v. 0%, *p* = .0039). The Hispanic population was under 5% in all four areas (4% in non-Appalachian metropolitan, 2% in non-Appalachian non-metropolitan, 1% in Appalachian metropolitan and non-metropolitan). Non-metropolitan and/or Appalachian regions had a significantly lower Hispanic population (*p* < .05), and within the non-metropolitan regions, the percentage of Hispanic residents was significantly lower than outside Appalachia (*p* = .0002). The percentage of those under the poverty level for non-Appalachian and Appalachian non-metropolitan counties (respectively, 16% ± 32% and 25% ± 44%) was significantly higher (both *p* < .05) than in non-Appalachian metropolitan ones (12% ± 38%).

Non-Appalachian non-metropolitan and Appalachian metropolitan counties had a comparable percentage of those under the federal poverty level (16% ± 32% and 16% ± 83%, respectively; *p* = .7103), and both were significantly lower than in Appalachian non-metropolitan counties (both *p* < .05).

Overall, non-Appalachian non-metropolitan and Appalachian metropolitan regions were demographically comparable. Appalachian metropolitan and non-metropolitan were comparable on all demographic factors except for poverty, where the Appalachian non-metropolitan region was 9 percentage points higher. Appalachian metropolitan was comparable to non-Appalachian metropolitan, except that the percentage of Hispanics was 3 points lower. Of the four regions, the non-Appalachian metropolitan was the most diverse in terms of race and ethnicity and had less population under the poverty level. On the other end of the spectrum, the Appalachian non-metropolitan region was the least diverse and with a higher population under the federal poverty level.

#### Mortality: All-cause, drug-related, and opioid-related

Over the 20-year study period (2000–2019), all-cause mortality in Kentucky decreased while drug- and opioid-related mortality both increased (Fig. 2). Drug- and opioid-related mortality rates were significantly higher in 2010–2019 compared to 2000–2009. All-cause age-adjusted mortality was comparable overall across the two decades (Fig. 2) and within each of the four regions (Table 2). The Appalachian non-metropolitan regions had the highest all-cause mortality of the four regions over the 20 years (Table 2). In the non-Appalachian regions, non-metropolitan counties had significantly higher all-cause mortality in 2010–2019 compared to metropolitan counties (950 ± 16 v. 883 ± 15; *p* = .0027), driving the difference observed over the 20 years and the difference in the changes in mortality across 2010–2019 v. 2000–2009 (Table 2).

Non-Appalachian metropolitan and non-metropolitan regions had comparable mortality related to drugs (both 18 per 100,000) and opioids (12 and 11 per 100,000, respectively) over 2000–2019. Similarly, Appalachian metropolitan and non-metropolitan regions had similar mortality attributable to drugs (29 and 30 per 100,000, respectively) and opioids (respectively, 22 and 21 per 100,000). There was a statistically significant difference, at about twice the rates, between the Appalachian and non-Appalachian regions (Table 2). Within each region, mortality due to drugs and opioids increased 59%–179% (*p* < .05) when comparing the periods 2010–2019 to 2000–2009. The changes were statistically comparable across all four regions.

## DISCUSSION

The results of this study suggest that the Appalachian (metropolitan and non-metropolitan) counties under study had higher all-cause, drug-related, and opioid-related mortality rates compared to non-Appalachian regions, with Appalachian non-metropolitan counties having the highest collective all-cause mortality rates.

The finding that the Appalachian counties under study had higher all-cause, drug- and opioid-related mortality than non-Appalachian regions is supported by the literature. The overall mortality rate in the U.S. from 1999 to 2014 declined by 10% but increased by 5% in the Appalachian Region.^12^ The primary abuse of opiates and synthetic drugs is of critical concern across Appalachia, as the hospital admission rates are higher in these regions than in the remainder of the U.S.^13^ Furthermore, drug and opioid abuse has been found to be most common among young, white males,^1^ and it has been documented that the Appalachian counties under study are less racially and ethnically diverse, with minority groups only making up 18.2% of the population, compared to 39% nationwide.^14^ Previous research has also identified socioeconomic factors—such as education, employment, and income—that are correlated to higher rates of opioid misuse.^12^ This study’s findings show that Appalachian non-metropolitan counties had the lowest diversity (lowest non-white, Hispanic) and highest households living under poverty level, statically different in composition from other regions. Therefore, the difference in mortality observed in Kentucky’s Appalachian non-metropolitan regions could be explained by these sociodemographic and economic differences. These issues are likely exacerbated by a confluence of factors in Appalachia, including the region’s reduced access to care for opioid abuse treatment,^15^ health disparities from widespread food deserts,^16^,^17^ and lower socioeconomic status (driven in part by high unemployment from the mining industry’s decline).^18^

The comparison of mortality between non-metropolitan and metropolitan Appalachia has not been explored in the literature. The findings in this study suggest that within the Appalachian portions of Kentucky, non-metropolitan counties have higher mortality, collectively, than metropolitan ones. These regions were demographically (i.e., in terms of sex, age, race, and ethnicity) comparable. However, the Appalachian non-metropolitan counties had a higher percentage of those living under the federal poverty level. Previous studies have linked higher poverty rates to increased mortality.^19^,^20^ In this study, Kentucky’s non-Appalachian non-metropolitan regions also had higher all-cause mortality than metropolitan regions in the period 2010–2019, a difference strong enough to be apparent in the combined years of 2000–2019. Additionally, non-metropolitan counties in this region had a higher poverty rate. Although there were additional demographic differences (age, race, and ethnicity), this similarity implies that the effects of rural v. urban health disparities^21^–^23^ could help explain higher mortality in Kentucky’s Appalachian non-metropolitan compared to metropolitan counties.

### Limitations

The results of this study should be considered alongside limitations associated with the nature of the data used. First, in the CDC WONDER data, some counties with low case count may be suppressed.^24^ Second, the American Community Survey is collected periodically and not in real time. Despite these limitations, the American Community Survey and CDC WONDER data are reliable sources of U.S. population and mortality data.

## IMPLICATIONS

The study demonstrates significant intra-state geographical disparities in Kentucky in terms of all-cause, drug-related, and opioid-related mortality, with the Appalachian counties having significantly higher rates of mortality compared to the non-Appalachian regions of Kentucky, both urban and rural.

The findings from this study can help identify trends in drug use in Kentucky to develop interventions and strategies to address the epidemic. Healthcare practitioners and public health officials should develop interventions addressing drug-related and opioid-related mortality in Kentucky targeted to the counties where mortality is significantly higher. Also, the information on geographic, demographic, and socioeconomic factors related to these mortalities can be factored into designing targeted interventions. Notably, programs addressing structural factors, such as access to opioid abuse treatment and poverty, should be considered. There is a need for integrated health services which not only focus on substance abuse prevention and treatment but also encourage culturally responsive medical care to the residents of the Appalachian Region.

**SUMMARY BOX**

**What is already known about this topic?**

Although drug overdose fatalities have risen nationally, some geographical regions have been disproportionately affected.

**What is added by this report?**

The study demonstrated significant intra-state geographical disparities in Kentucky in all-cause, drug-related, and opioid-related mortality, with the Appalachian region having significantly higher rates when compared to the non-Appalachian regions of Kentucky.

**What are the implications for future research?**

The findings from this study will be beneficial in identifying trends of drug use in Kentucky to develop interventions and strategies which could shift the substance use epidemic in the future. Also, the information on geographic, demographic, and socioeconomic factors related to these types of mortality can be factored into the interventions’ design specific to targeting population’s sociodemographics.

## Figures and Tables

**Table 1:** Demographics and socioeconomics of KY counties using 2010 population estimates

2010 Population estimate (% ± SD)		Non-Appalachian		Appalachian		*p*-value					
		Metro [1]	Non-Metro [2]	Metro [3]	Non-Metro [4]						
		*n = 2,317,627*	*n = 785,562*	*n = 133,683*	*n = 1,048,956*	[2] vs [1]	[3] vs [1]	[4] vs [1]	[3] vs [2]	[4] vs [2]	[4] vs [3]
**Sex**	**Males**	49 ± 6	49 ± 10	49 ± 32	50 ± 9	.9207	.9809	.7043	.9821	.6136	.8485
	**Females**	51 ± 6	51 ± 10	51 ± 32	50 ± 9	.9217	.9812	.7052	.9823	.6155	.8485
**Age**	**0–17**	24 ± 2	23 ± 4	23 ± 8	23 ± 3	.1026	.3475	.0871	.8471	.9544	.8206
	**18–65**	64 ± 4	61 ± 6	62 ± 14	63 ± 6	.4661	.8171	.9638	.9144	.4427	.8285
	**65+**	12 ± 2	15 ± 3	16 ± 10	14 ± 2	.0004	.0685	.0163	.9589	.1214	.4691
**Race**	**White**	85 ± 60	93 ± 25	96 ± 62	97 ± 19	.7405	.4467	.0210	.5422	.0416	.7968
	**Black**	12 ± 10	6 ± 5	3 ± 8	2 ± 2	.7744	.1898	< .0001	.1501	< .0001	.2281
	**AIAN**	1 ± 2	1 ± 1	1 ± 2	1 ± 2	.5186	.7555	.3156	.9799	.7565	.9147
	**Asian**	2 ± 2	1 ± 1	1 ± 2	0 ± 1	.0960	.4066	.0039	.8707	.2402	.7299
	**NHOPI**	0.13 ± 0.42	0.07 ± 0.36	0.04 ± 0.22	0.06 ± 0.22	.7206	.8241	.6155	.9391	.9092	.9749
	**Other**	2 ± 6.724	1 ± 1.927	0 ± 2.507	1 ± 1.518	.0493	.1881	.0013	.5089	.2345	.8110
**Ethnicity**	**Hispanic**	4 ± 131	2 ± 12	1 ± 17	1 ± 7	.013	.0438	< .0001	.2285	.0002	.8094
	**Below FPL**	12 ± 38	16 ± 32	16 ± 83	25 ± 44	.0237	.1394	< .0001	.7103	< .0001	.0004

NOTES: Entries are percent estimates with their standard deviations. The following abbreviations are used within the table: **FPL:** Federal poverty level; **Metro:** Metropolitan; **Non-Metro:** Non-Metropolitan; **AIAN:** American Indian and Alaska Native; **NHOPI:** Native Hawaiian and Other Pacific Islander

**Table 2:** All-cause, drug-related, and opioid-related mortality, 1999–2019

		Non-Appalachian		Appalachian							
Mortality*		Metro [1]	Non-Metro [2]	Metro [3]	Non-Metro [4]	*p*-values†					
						[2] vs [1]	[3] vs [1]	[4] vs [1]	[3] vs [2]	[4] vs [2]	[4] vs [3]
**All-cause mortality**	**2000–2019**	904±11	959±11	938±32	1076±10	0.0006	0.3127	<.0001	0.5402	<.0001	0.0002
	**’00–’09**	926±16	969±16	942±46	1072±15	0.0586	0.7294	<.0001	0.5901	<.0001	0.0114
	**’10–’19**	883±15	950±16	934±45	1079±15	0.0027	0.2794	<.0001	0.7431	<.0001	0.0045
	**Ratio**	0.95±1.02	0.98±1.02	0.99±1.07	1.01±1.02	0.03476	0.07566	0.03303	0.07311	0.03092	0.07234
	***p*** **-value**	0.0585	0.3963	0.8971	0.7041						
**Drug-related mortality**	**2000–2019**	18±1	18±1	29±4	30±1	0.5549	0.0036	<.0001	0.0012	<.0001	0.748
	**’00–’09**	12±1	13±1	23±5	24±1	0.8246	0.0097	<.0001	0.0133	<.0001	0.799
	**’10–’19**	28±2	24±2	37±6	38±2	0.1822	0.1478	0.0006	0.0317	<.0001	0.8445
	**Ratio**	2.29±1.13	1.94±1.13	1.62±1.32	1.59±1.09	0.1466	0.2134	0.1026	0.2507	0.119	0.2816
	***p*** **-value**	<.0001	<.0001	0.0825	<.0001						
**Opioid-related mortality**	**2000–2019**	12±1	11±2	22±4	21±1	0.5687	0.006	<.0001	0.006	0.0003	0.8428
	**’00–’09**	7±1	7±2	15±4	16±1	0.9403	0.0341	<.0001	0.0873	0.0143	0.74
	**’10–’19**	21±2	17±2	33±8	28±2	0.1666	0.0743	0.0113	0.0142	0.0002	0.5052
	**Ratio**	2.79±1.21	2.37±1.38	2.26±1.43	1.74±1.12	0.3191	0.3302	0.1383	0.4615	0.2515	0.2911
	***p*** **-value**	<.0001	0.0087	0.0257	<.0001						

DATA SOURCE: CDC WONDER

NOTES:

* Values are rates per 100,000 adjusted for age, sex, race, ethnicity, and poverty-level and associated standard errors.

† *P*-values are from multivariable negative binomial distribution linear contrasts.
